# The Impact of the COVID-19 Lockdown “Home Quarantine” on the Physical Activity and Lifestyle of Children in Qatar

**DOI:** 10.3389/fpubh.2022.877424

**Published:** 2022-05-25

**Authors:** Nada A. Al-Mulla, Ziyad R. Mahfoud

**Affiliations:** ^1^Department of Medical Education, Weill Cornell Medicine - Qatar, Doha, Qatar; ^2^Division of Epidemiology, Department of Population Health Sciences, Weill Cornell Medicine, New York, NY, United States

**Keywords:** COVID-19, quarantine, lockdown, physical activity, lifestyle, children, Qatar

## Abstract

**Background:**

Several studies have investigated how the coronavirus disease 2019 (COVID-19) pandemic impacted children's lifestyle. To our knowledge, this is the first study that assesses the impact of quarantine on physical activity, screen time, sleep, and diet in children aged 5 to 12 in Qatar.

**Methods:**

Cross-sectional data from an online survey distributed in Qatar was analyzed. The survey measured the parents' or caregivers' assessment on the change in the child's physical activity, sleep, screen time, and diet between the two periods (before quarantine and during quarantine). The data was analyzed using frequency distributions, paired t-test and McNemar's test.

**Results:**

Data from 144 respondents were analyzed. Due to the quarantine, the total weekly average hours of physical activity significantly decreased with a greatest reduction for the school and after school durations. Only 4.5% of the children were engaging in at least 60 minutes of physical activity per day (in contrast to 25.6% prior to quarantine). The reported barriers for physical activity were screen time for school (52.8%) and leisure (51.4%). There was a significant increase in the total number of main meals per day, with a higher consumption of unhealthy food. The majority of the children had their bedtime and waketime shifted to later because of the quarantine. The parents' or caregivers' satisfaction with the child's lifestyle during quarantine showed that 49.1% were disappointed or very disappointed. Also, 53.8% described their child's mental health as “better before quarantine.”

**Conclusions:**

Quarantine had a negative impact on the lifestyle of children in Qatar. When implementing restrictions, authorities should consider some interventions to counterpart such impact.

## Introduction

The coronavirus disease 2019 (COVID-19) pandemic spread globally and infected millions of people worldwide leading to hospitalizations and death (1, 2). The global response to COVID-19 was a declaration to implement an emergency lockdown in early March 2020 (2). To prevent transmission, and with the aim to encourage social distancing, governments issued mandatory closure of schools following the policy of the U.S. Centers for Disease Control and Preventions (CDC) (3). Other public areas such as parks, playgrounds and recreational facilities were also closed (4). Only essential outings such as grocery shopping or pharmacy visits were allowed. Despite the slow reopening, it remains uncertain when schools and other public places will lift all restrictions, as such decisions depend on how the pandemic evolves (5).

The behavioral adaptations caused by quarantining impacted the daily lifestyle and health of children (6–8). While staying at home during the pandemic is a safe measure to avoid transmission of the virus, prolonged stays at home can lead to physical inactivity (9–11). Schools are considered a primary resource for children's physical activity, and thus mandated closures limited the opportunities for physical activity (e.g., physical education classes, after school sports) (12, 13). Another essential resource for physical activity is outdoor play areas, which were inaccessible during quarantine (14, 15). Physical activity was limited to virtual options provided by schools or activity encouraged at home by the family. Stay-at-home orders not only affected physical activity levels, but also other lifestyle factors such as increased screen time (16, 17), increased intake of unhealthy food (18, 19), disrupted sleep patterns (20), and had a negative impact on children's mental health (21, 22).

Recommendations for children and adolescents indicate that they should accumulate >60 min of moderate to vigorous intensity physical activity (23). Despite these recommendations, international epidemiological data from 2016 shows that <10% of children and adolescents achieved the daily physical activity recommendations (24). Another study that objectively assessed the physical activity level from school children in Qatar showed that <15% engage in moderate to vigorous activity during school hours and the majority spend them in sedentary behaviors (13). The coronavirus remains a global threat, and this has added a unique challenge for children to meet the physical activity recommendations and maintain a healthy lifestyle routine.

The long duration of school closure and decreased physical activity strongly predict increases in Body Mass Index (BMI) and the prevalence of childhood obesity (25). Consequently, children who are overweight or obese at age 5 are more likely to develop higher BMI at age 50 (26). Physical inactivity and the increase in sedentary lifestyle in childhood is a public health concern, as such children are at increased risk for developing non-communicable diseases like obesity, type 2 diabetes, and cardiovascular diseases in adulthood as well as mental disorders such as anxiety and depression (27–33).

Thus, it is imperative to understand the changes in lifestyle behaviors, particularly physical activity, due to the COVID-19 pandemic. To date, there are insufficient data that demonstrate the impact of the COVID-19 outbreak on physical activity, screen time, diet, and sleep in children aged 5–12. The present study aims to test for changes in physical activity in children due the COVID-19 pandemic and explore the potential associated influencing factors. Changes in screen time, diet, and sleep due to the quarantine will also be assessed. The study results will increase awareness of the impact of the pandemic on children's health and wellbeing.

## Methods

### Study Design and Sample

The research team collected cross-sectional data using convenience sampling *via* an anonymous online questionnaire. Qualtrics (Qualtrics, Provo, UT) was used to create the questionnaire and collect the data. Based on pilot testing, the time to complete the questionnaire was estimated to be 5–10 min. The questionnaire was first created in English, and then an Arabic version was developed by a certified translator from Weill Cornell Medicine in Qatar (WCM-Q) and piloted for comprehension by five bilingual people. No major edits were needed. Consequently, people who consented to participate had the option to complete the survey in either English or Arabic.

A message was distributed by email and posted on social media platforms such as (Facebook, WhatsApp, etc.) that described the survey, including the eligibility criteria, voluntary nature of participation, participant anonymity (no identifiers were collected), and request for consent with a link. Facebook and WhatsApp groups with interest in school-aged children were targeted. Participants who responded to the survey were considered to have given consent, and this was explained in the message as described previously. Data collection began in January 2021 and continued until June 2021 during Qatar's implementation of quarantine, when schools, worksites, and public places were closed, and residents were asked to practice social distancing during essential outings. To be eligible for the study, participants had to be (1) at least 18 years old (2) caregivers or parents taking care of a child between the ages of 5 and 12, and (3) able to understand either English or Arabic. This study was approved by the WCM-Q Institutional Review Board (# 1632824-2).

### Measures

The survey instrument included a total of 23 questions that were divided into 4 sections. Section Introduction included demographic questions about the child and the parent or caregiver. Section Methods included questions about the child's physical activity, changes during the quarantine period, and potential associated environmental or social factors that might have influenced this change. Section Results provided similar questions related to other lifestyle habits, including change in child's screen time, dietary habits, and sleep. Section Discussion included questions related to the parents' or caregivers' satisfaction of the effect of the quarantine on the child's lifestyle, family practices of social distancing, and the impact of quarantine on the child's mental health wellbeing. To measure change, participants were asked to answer questions measuring the child's physical activity, sleep, screen time and dietary behavior for the period before and the period during quarantine. The full version of the questionnaire is available in Supplementary Data Sheet 1.

### Sample Size

With at least 130 participants, the study has an 80% power to test an effect size of 0.25 standard deviation using the paired *t*-test.

### Statistical Analysis

The data was extracted from Qualtrics into Microsoft Excel in June 2021. A total of (*n* = 366) entries were obtained, screened and cleaned. Participants who failed to meet the eligibility criteria were excluded (*n* = 41). Also, participants who only answered demographic questions (*n* = 181) were excluded. The final sample consisted of (*n* = 144) respondents.

Variables were summarized using frequency distributions [number (n) and percentage (%)] for categorical variables such as the child's gender and means ± standard deviations as well as medians for numeric variables like age. BMI (in Kg/m2) was calculated based on the height and weight of each child and then categorized based on the CDC's gender-specific BMI-for-age percentiles (23). Children were then classified into underweight, healthy weight, overweight and obese.

The main analysis included assessing changes in the average duration of physical activity per week between the two periods (before quarantine and during quarantine) using the paired *t*-test. The changes in meals per day and average sleep per night were assessed similarly. The McNemar's test was used to compare the changes in physical activity between the two time periods. Changes in other lifestyle factors, such as the change in screen time and the amount of healthy or unhealthy food, were analyzed using the McNemar's test. Questions about satisfaction, mental health and family behavior during quarantine were summarized using frequency distributions.

IBM-SPSS (version 27 Armonk, NY) was used for all the analyses. For each variable, the number of missing data was reported. A *p*-value of 0.05 or lower was considered statistically significant.

## Results

### Demographics

Participants' demographics are summarized in Table 1. The average age of the children the participants care for was 8.1 ± 2.1 years and ranged from 5 to 12 years. Most children were males (57.7%) and the oldest sibling (52.8%). Qatari (46.5%) was the most reported nationality and almost half of the children had a healthy weight (48.5%), as per the CDC growth chart. The majority of the caregivers surveyed were mothers (80.3%), ranging in age from 30 to 39 years old (39.4%) and had a bachelor's degree (56.7%).

**Table 1:** Descriptive data for demographic characteristics.

**Child age, in years**	
Mean ± SD	8.1 ± 2.1
Median (Q1–Q3)	8.0 (6.0–10.0)
Min-max	5.0-12.0
**Child gender*, n* (%)**	
Female	60 (42.3)
Male	82 (57.7)
Missing	2
**Nationality*, n* (%)**	
Qatari	66 (46.5)
Other Arabs	14 (9.9)
European/American/Canadian	44 (31.0)
Rest of the world (Asians)	18 (12.7)
Missing	2
**BMI category*, n* (%)**	
Underweight	9 (8.7)
Healthy weight	50 (48.5)
Overweight	17 (16.5)
Obese	27 (26.2)
Missing	41
**Rank of child among siblings*, n* (%)**	
Oldest	75 (52.8)
Middle	15 (10.6)
Youngest	52 (36.6)
Missing	2
Relationship to the child*, n* (%)	
Mother	114 (80.3)
Father	11 (7.7)
Others (Sibling, Aunt, Uncle, Grandparents)	17 (12.0)
Missing	2
**Age of parent or caregiver*, n* (%)**	
<20	4 (2.8)
20–29	9 (6.3)
30–39	56 (39.4)
40–49	53 (37.3)
≥50	20 (14.1)
Missing	2
**Level of education of parent or caregiver*, n* (%)**	
Middle school	2 (1.4)
High school	6 (4.3)
Bachelors	80 (56.7)
Masters	39 (27.7)
PhD/other	14 (9.9)
Missing	3

### Physical Activity and Associated Factors

**Figure 1:**
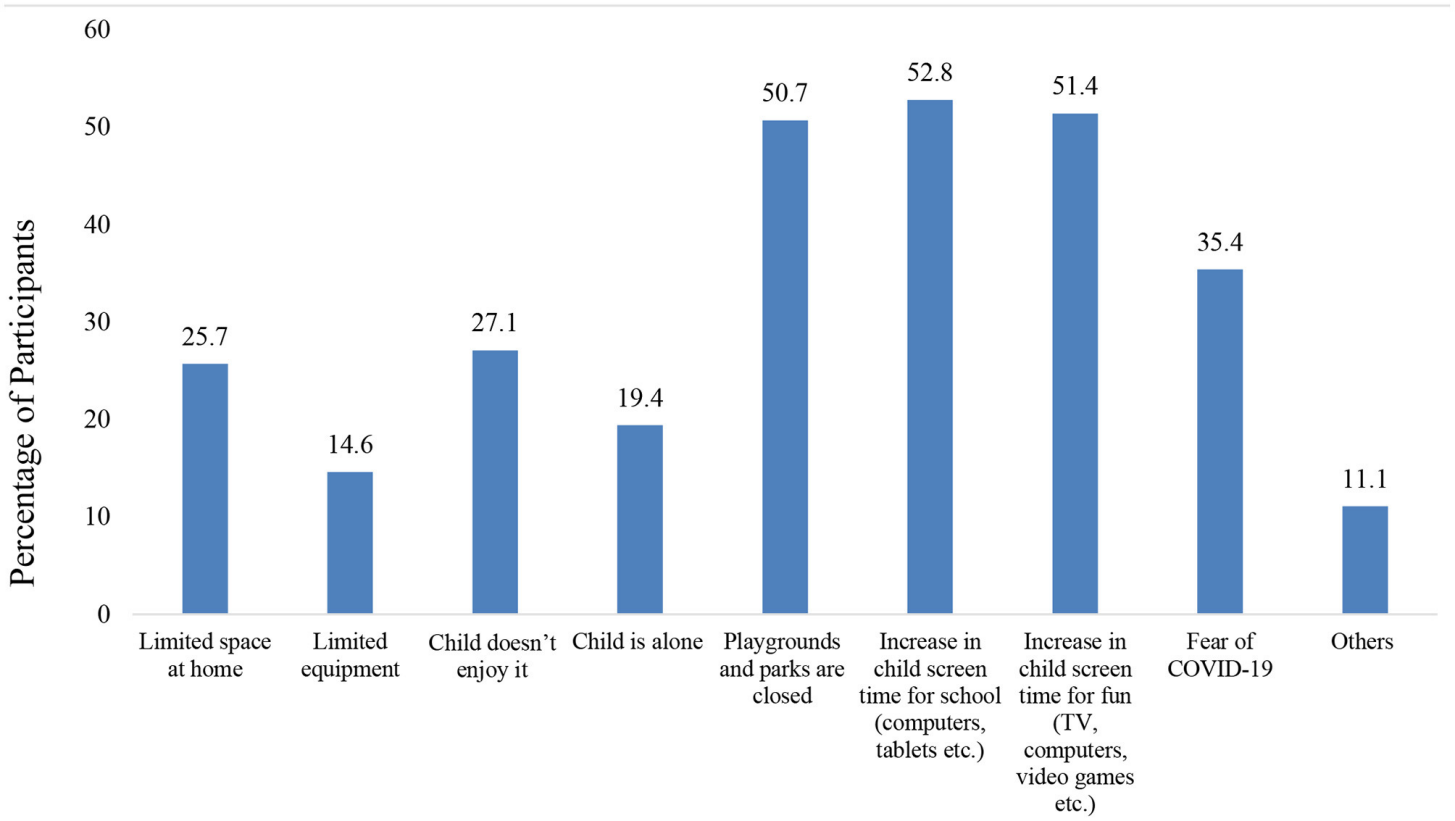
Factors that decreased the child's physical activity during quarantine.

**Figure 2:**
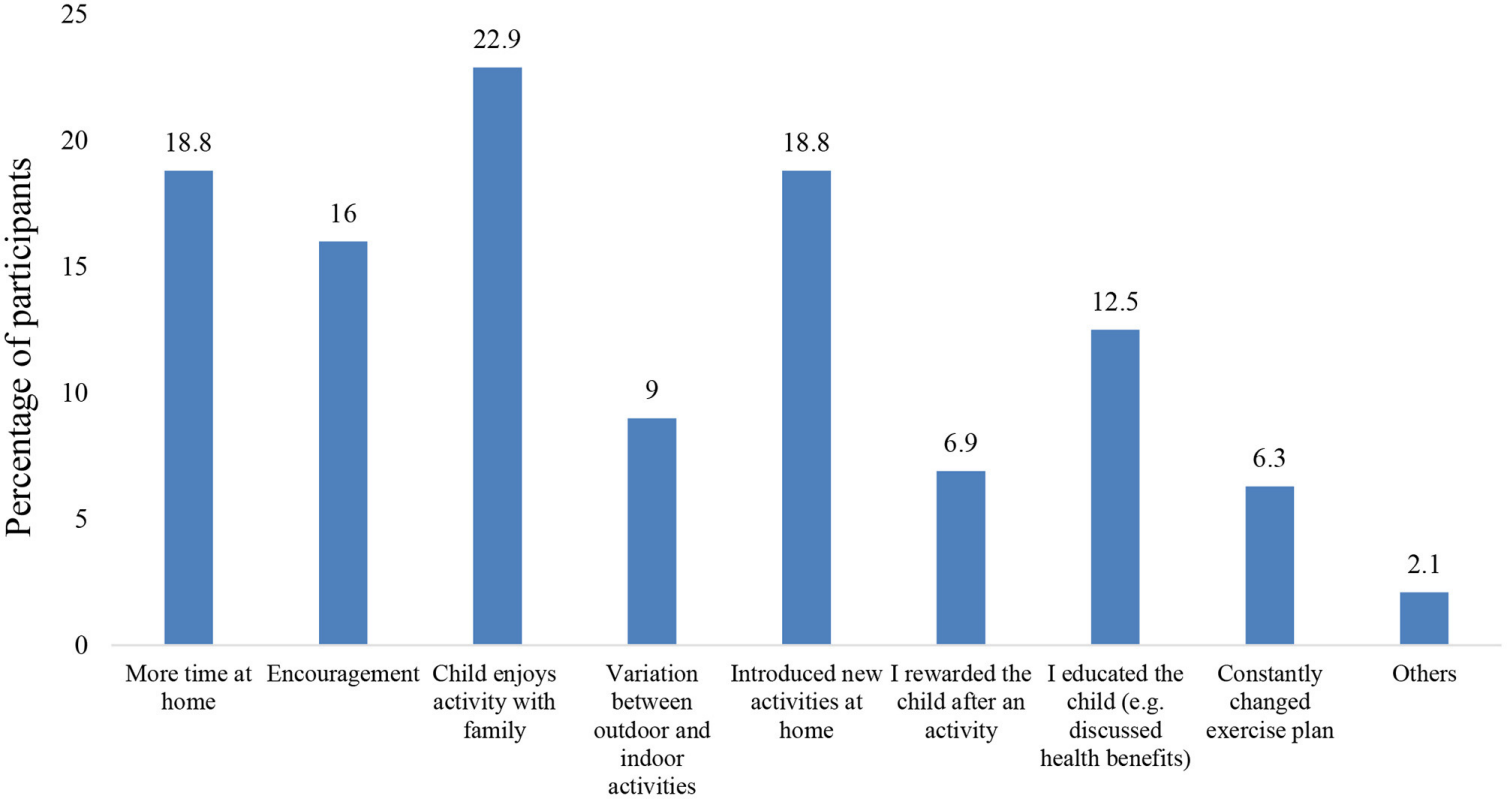
Factors that increased the child's physical activity during quarantine.

Due to the quarantine, at school and after school weekly average hours of physical activity significantly decreased by 3.5 and 2.2 h respectively. Also, there was a decrease of 0.84 h at home, but this was not statistically significant (see Table 2). Before the quarantine, the majority of parents or caregivers (63.9%) reported that their children had a high (5–7 h/week) or very high (>7 h/week) activity level. However, during the quarantine, 48.9% reported that their children had minimal activity (<2 hours/week) or none, which represents a statistically significant change (*p* < 0.01) (see Table 2). Most parents or caregivers reported that the factors that lead to the decrease in physical activity during quarantine were screen time for school (52.8%), screen time for leisure (51.4%), and park closure (50.7%) (see Figure 1). In contrast, the parents or caregivers who reported increased physical activity during quarantine attributed it to factors like the child enjoying it with family (22.9%), spending more time at home (18.8%), and introducing new activities at home (18.8%) (see Figure 2).

**Table 2:** Comparison of the physical activity, screen time, dietary habits, and sleep before quarantine and during quarantine.

	**Before quarantine**	**During quarantine**	***P*-value**
**Average hours of physical activity per week**, (mean ± SD)			
At school	5.4 ± 6.9	1.9 ± 3.9	<0.01
After school	3.3 ± 4.6	1.1 ± 2.5	<0.01
At home	3.9 ± 5.6	3.1 ± 4.1	0.066
Overall	12.5 ± 14.1	6.0 ± 7.0	<0.01
**Physical activity**, [*n* (%)]			
Very high (>7 h/week)	34 (25.6)	6 (4.5)	<0.01
High (5–7 h/week)	51 (38.3)	13 (9.8)	
Moderate (2–4 h/week)	42 (31.6)	49 (36.8)	
Minimal (<2 h/week)	5 (3.8)	54 (40.6)	
None (0 h/week)	1 (0.8)	11 (8.3)	
Missing	11	11	
**Screen time (hours/day)**, [*n* (%)]			<0.01
0–1 h	42 (39.6)	2 (1.9)	
2–3 h	53 (50.0)	25 (23.6)	
4–5 h	7 (6.6)	39 (36.8)	
6–7 h	2 (1.9)	23 (21.7)	
≥8 h	2 (1.9)	17 (16.0)	
Missing	38	38	
**Meals per day**, (mean ± SD)	3.28 ± 0.7	3.81 ± 1.2	<0.01
**Amount of healthy food**, [*n* (%)]			0.156
Never	1 (1.0)	4 (3.8)	
Once/week	8 (7.6)	10 (9.6)	
2–4 days/week	29 (27.6)	27 (26.0)	
5–6 days/week	22 (21.0)	17 (16.3)	
Everyday	30 (28.6)	26 (25.0)	
More than once a day	15 (14.3)	20 (19.2)	
Missing	39	40	
Amount of unhealthy food, [*n* (%)]			<0.01
Never	2 (1.9)	11 (10.4)	
Once/week	57 (53.8)	22 (20.8)	
2–4 days/week	34 (32.1)	44 (41.5)	
5–6 days/week	6 (5.7)	9 (8.5)	
Everyday	5 (4.7)	12 (11.3)	
More than once a day	2 (1.9)	8 (7.5)	
Missing	38	38	
**Average sleep duration per night**, (mean ± SD)	9.1 ± 1.4	8.9 ± 2.1	0.354

### Screen Time, Diet, and Sleep

Prior to the quarantine, 10.4% of the parents or caregivers reported that the child spent at least 4 hours per day using screen time for leisure (e.g. TV, video-games, smart phones or tablets), but during the quarantine, this increased to 74.5% (*p* < 0.01) (see Table 2).

There was a significant increase (*p* < 0.01) in the number of main meals per day from 3.28 prior to quarantine to 3.81 during quarantine. Furthermore, the results indicate that there were no significant changes (*p* > 0.05) in the overall amount of healthy food, such as fruits, vegetables, and whole grains, consumed before and during the quarantine. By contrast, the amount of unhealthy food (like fast food, soft drinks, and sweets) increased significantly (*p* < 0.01). Before quarantine, the majority consumed unhealthy meals on 1 or less day per week (54.7%) whereas during quarantine the majority (69%) consumed unhealthy meals on 2 days per week or more (see Table 2).

**Figure 3:**
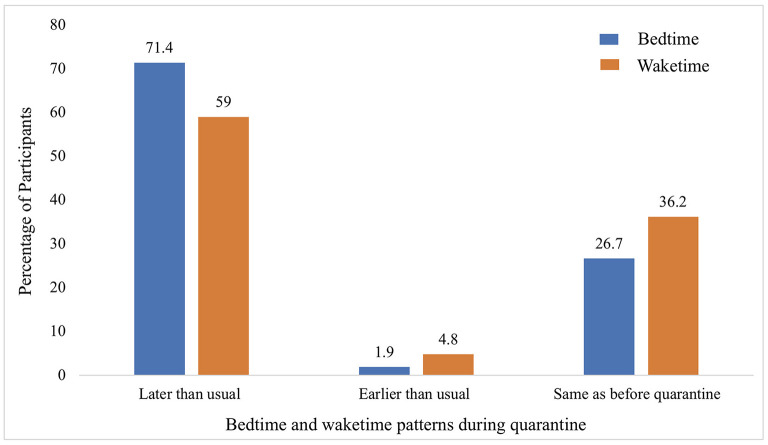
Changes in sleep patterns during quarantine.

There was no significant change (*p*>0.05) in the average sleep duration per night between prior to quarantine (9.1 h) and during quarantine (8.9 h) (see Table 2). However, due to the quarantine, sleep patterns for most children changed, with 71% having bedtime later than usual and 59% having their waketime later than usual (see Figure 3).

### Overall Lifestyle, Mental Health and Adherence to Social Distancing

**Figure 4:**
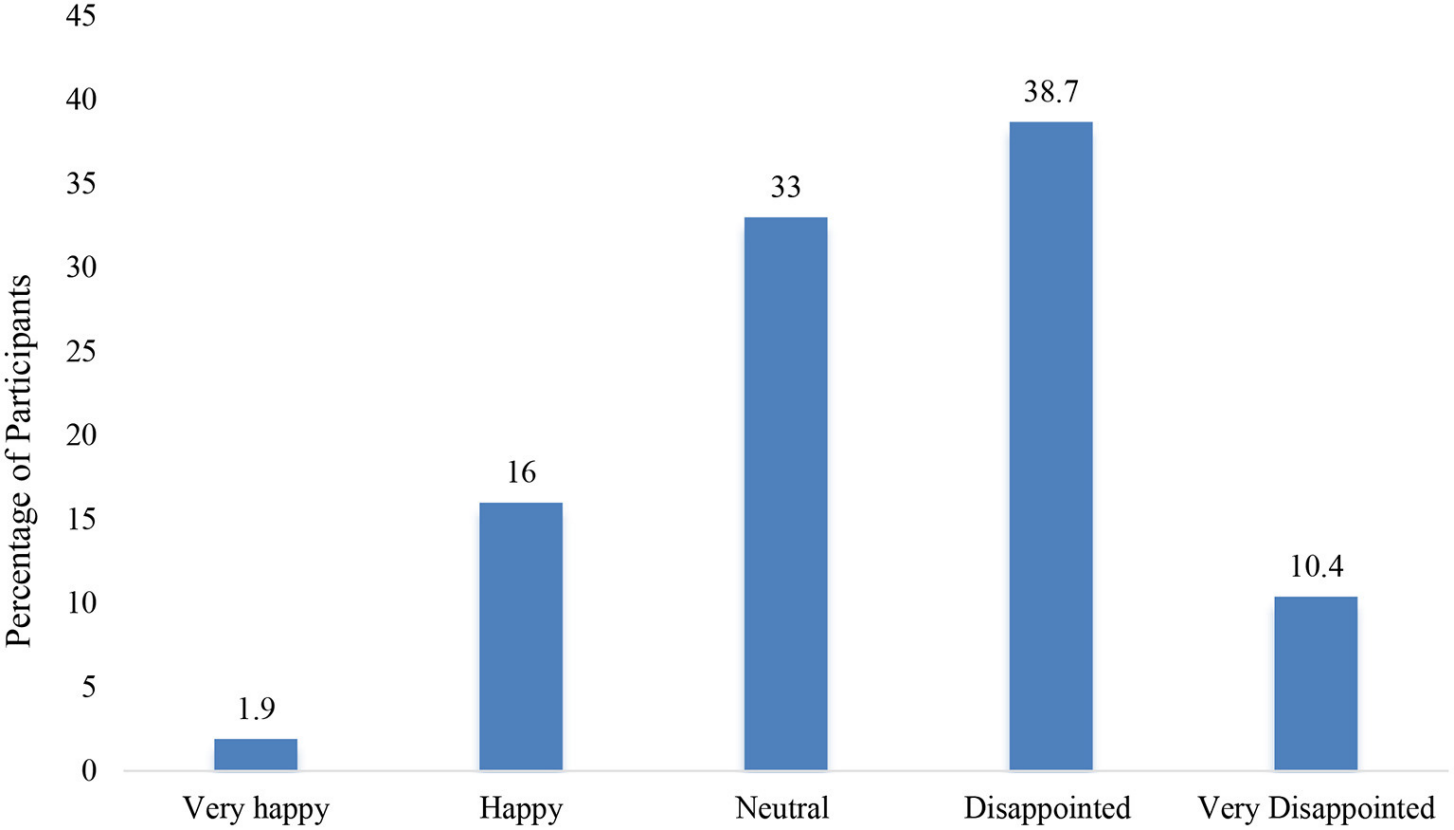
Overall satisfaction of the caregiver or parent with the child's lifestyle during quarantine (this includes physical activity, sleep, diet, and screen time).

**Figure 5:**
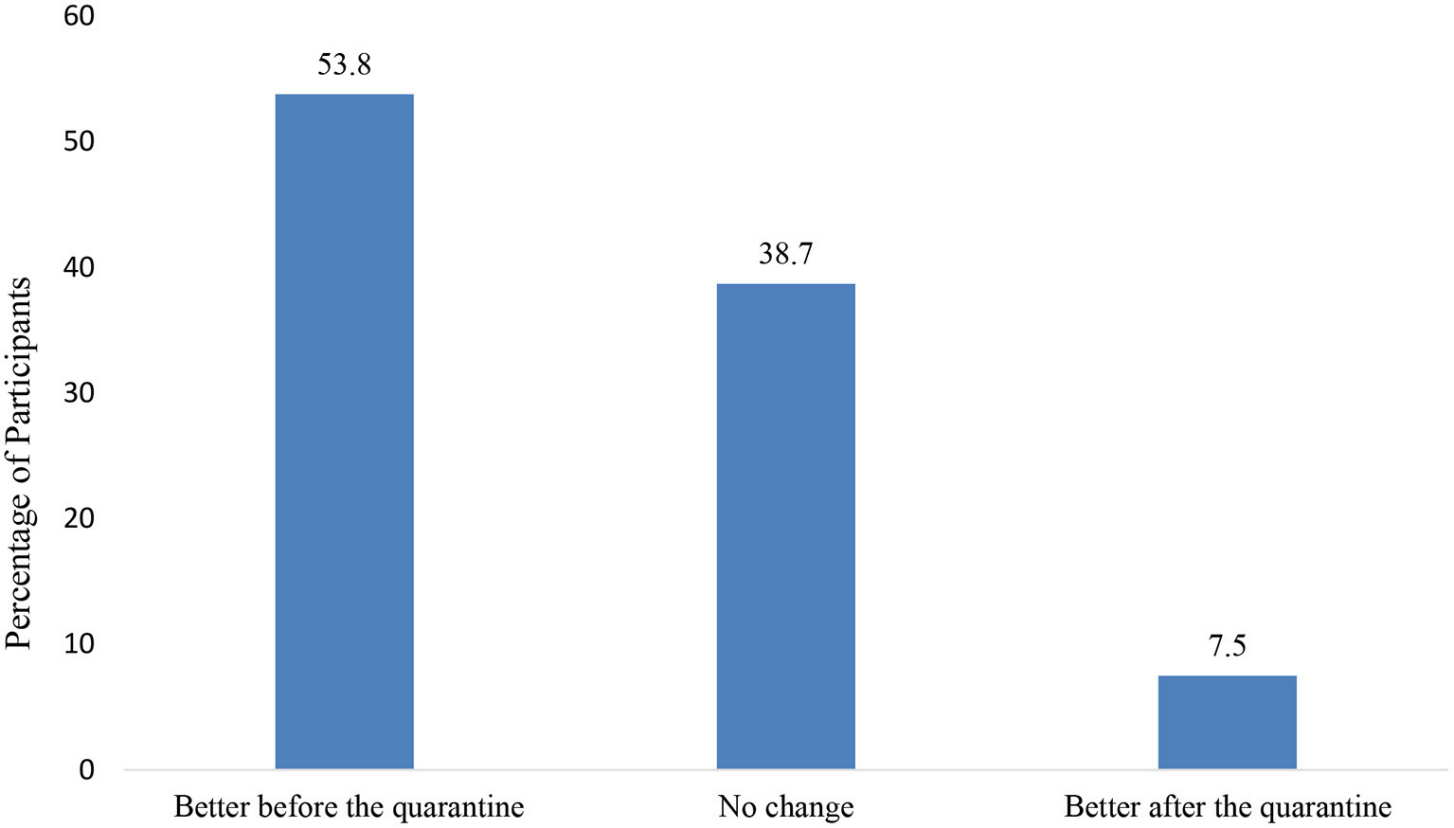
Caregiver or parent rating the mental health wellbeing of the child when comparing it between before and during quarantine.

**Figure 6:**
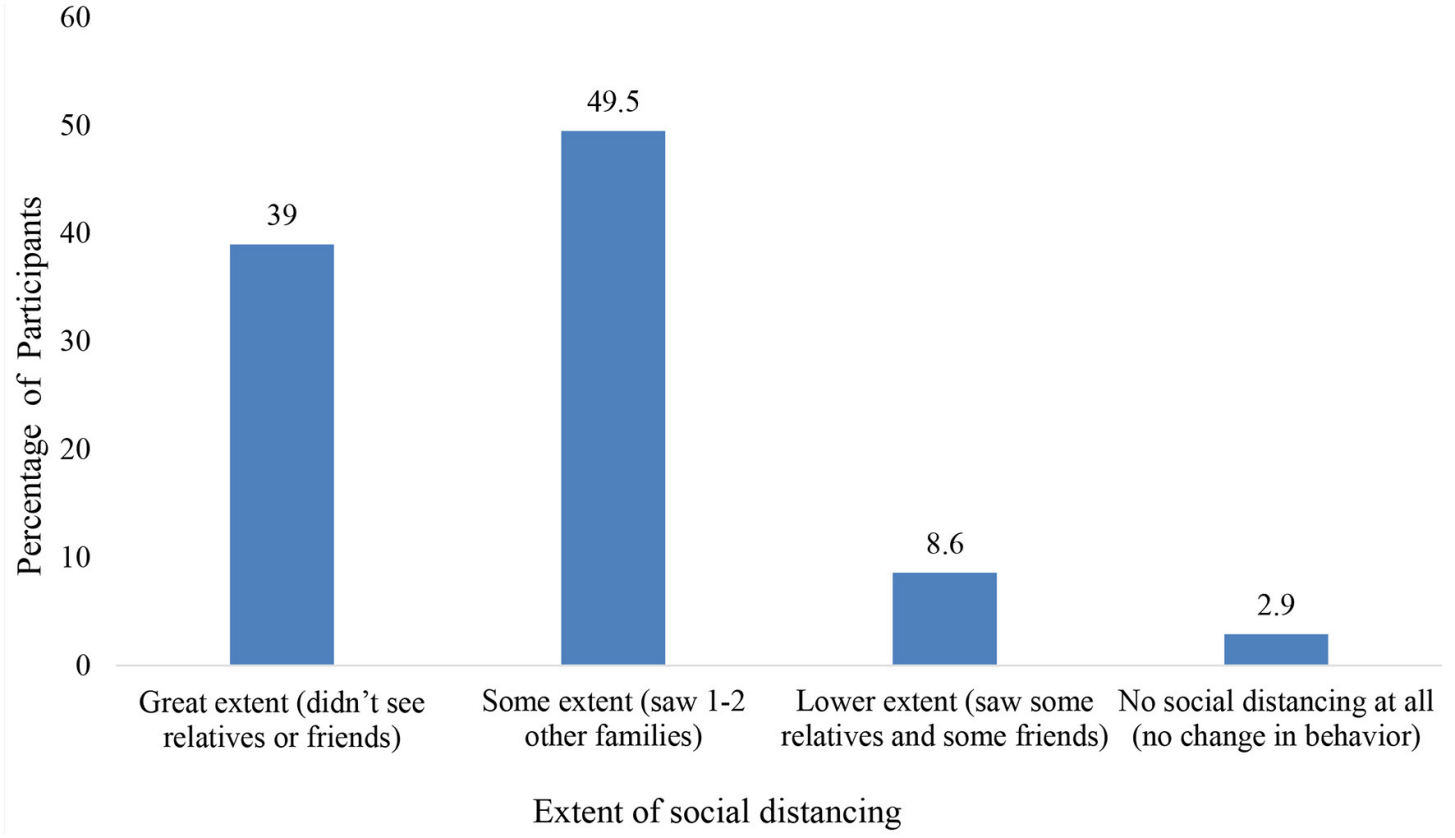
Caregivers' or parents' perception of the family's social distancing behavior during quarantine.

The result for the overall satisfaction of the parent or caregiver with the child's lifestyle showed that 49.1% were disappointed or very disappointed about their child's lifestyle during the quarantine (this includes physical activity, sleep, diet, and screen time) (see Figure 4). Also, the majority of the parents or caregivers (53.8%) rated that their child's mental health as better before quarantine and only 38.7 % reported no change (see Figure 5). Moreover, 88.5% of parents or caregivers reported that their family practiced social distancing to some extent (saw 1–2 families) or to a great extent (didn't see anyone) (see Figure 6).

## Discussion

This study represents novel data from a diverse sample in Qatar that explores the parents' or caregivers' assessment about the changes in physical activity, screen time, sleep and dietary behavior in school-aged children during the COVID-19 pandemic. To our knowledge, this is the first study conducted in Qatar that assesses the impact of the quarantine on various health behaviors among children aged 5–12.

Due to the quarantine, the total weekly average hours of physical activity significantly decreased. The reduction in physical activity was greatest for the school and after school durations. Support for this finding comes from a longitudinal study that objectively measured the impact of school closure due to the pandemic on physical activity, and this showed that the greatest reduction was from 8:00am to 7:00 pm, the times when children would have been engaged in in-person school and after school activity (20). Additionally, our study shows that only 4.5% of these children were engaging in the recommended 60 min of physical activity per day, which is considerably lower than the pre-quarantine estimate of 25.6%. This is consistent with other studies that also reported a significant downward trend in the amount of physical activity in children caused by quarantine (10, 11, 17, 34–37). In particular, a study that explored the impact of COVID-19 on physical activity in Canadians showed that 3.6% of children and 2.6% of adolescents (10) were achieving the physical activity recommendation during the pandemic, compared to 12.7% meeting the guidelines in 2019, prior to the pandemic (38).

There are several studies that explore the physical and mental health consequence of quarantine. Specifically, there is emerging evidence related to COVID-19 negatively impacting children's mental health with reported increases in stress, anxiety, loneliness, frustration, and hyperactivity (8). This resonates with our study, which found that most parents and caregivers rated that their child's mental health as better before the quarantine. Furthermore, another systematic review has shown that physical inactivity and school closure contributed to childhood obesity and an increased BMI (19). The prevalence of overweight/obesity observed in this study was (42.7%), which is consistent with previous estimates from Qatar (39, 40). Being obese adds an additional risk factor for negative lifestyle changes and weight gain during quarantine and may worsen clinical outcomes when infected with COVID-19 (41).

In addition to the impact of school closure on physical inactivity, the increase in sedentary behavior at home and inaccessibility to outdoor areas had a significant influence on the reduction of physical activity. In fact, the majority of parents and caregivers reported that screen time for school/leisure and closure of parks served as barriers for maintaining physical activity. The prevalence of screen time for at least 4 h per day was 74.5% (in contrast to 10.4% prior to quarantine). This is consistent with recent studies from different countries that reported an increase in sedentary behavior during the pandemic (17, 42–44). According to a recent systemic review, there is a variety of negative health effects of increase in screen time in children, with strong evidence of association with obesity, depression, unhealthy diet and poorer quality of life (45). Also, recent studies have shown that the prevalence of myopia in children increased during the pandemic (46, 47). Specifically, there was a positive association between online time and increase in myopia incidence (47).

Furthermore, our study found that there was a significant increase in the total number of main meals per day, with higher consumption of unhealthy food (fast food, soft drinks, sweets, etc.,). This is consistent with a review of 15 articles, which concluded that dietary behaviors deteriorated due to the pandemic (19). The review showed that there was an increase in consumption of fried food, and sweets, which was associated with a higher BMI. Similarly, a study based on an international survey conducted during quarantine showed a significant increase in consuming unhealthy food, eating out of control, snacking between meals and in the total number of meals (18). This study showed no significant changes in healthy food intake, which is similar to a Spanish study that reported no statistical difference in fruit and vegetable consumption in children aged 6 to 16 (43). Furthermore, a review by Cena et al. (41) gathered multiple studies from different countries (e.g., China, Palestine, USA, Poland, and Italy) to show that unhealthy weight gain in children during the pandemic was associated with increased consumption of unhealthy food. This becomes relevant since a healthy diet improves physical and mental health, as shown in prior studies conducted in epidemics (48).

In this study, there was no significant change in sleep duration; however, most of the children had their bedtime and waketime shifted to later because of the quarantine. A recent systematic review that combined 71 studies from 35 different countries to explore the impact of the restrictions among children and adolescents reported discrepancies on the effect of the pandemic on sleep (49). Nevertheless, our findings correlated with 11 studies that showed no significant change in sleep duration, and 10 other studies that reported a shift to a later bedtime and wake time (49). Clearly, the stresses brought by the pandemic encouraged a shift toward an evening oriented circadian rhythm. Light exposure during daytime is essential for maintaining circadian regulation (50), and this was subjected to disruption due to home confinement. In fact, based on parental perception, the most significant factors that affected their children's sleep routine were no traveling to/from school, early school start time and increase in Internet time (51). The increase in blue light from screen time near bedtime can dampen the release of melatonin and further contribute to a delay in sleep latency (52). Therefore, it is vital to establish a structured sleep routine as it has significant implications for behavioral (e.g., conduct and attention) and emotional regulation (e.g., depression and anxiety) (53).

This study has several limitations that should be considered when interpreting the results. First, the data was collected from a convenience sample using a self-reported questionnaire from parents or caregivers on the change in the child's behavior caused by the quarantine. This is subject to recall and social desirability biases that weaken the generalizability of the results. Second, although the sample was from the general population, the sample size was small, so the results should be interpreted with caution. Third, incomplete responses or missing data hindered the statistical analysis to be underpowered for few comparisons. Fourth, our definition of physical activity may not have covered all activities that children participate in. Fifth, as this is a voluntary online survey, self-selection bias is another potential limitation of the study and limits the generalizability of the results. Finally, this is a cross-sectional study, which may not allow for the interpretation of causal relationships between variables. Despite these limitations, this study is first of its kinds and provides new insight into the impact of quarantine due the COVID-19 pandemic on the wellbeing of children in Qatar. The sample is diverse, covering children of different ages and gender. This can serve as valuable data to be shared with the scientific community and to track health changes for future studies.

In conclusion, this study examined the changes in physical activity, screen time, diet, and sleep among children in Qatar as reported by the parent or caregiver. We found that under COVID-19 restrictions children are less physically active, spend more time on screens, adopt unhealthy dietary behaviors, and have later bedtimes and waketimes compared to before the quarantine. The overall satisfaction of the parents or caregivers regarding the child's lifestyle showed that about half of them were disappointed or very disappointed about their child's lifestyle behaviors during the quarantine. The majority described that their children's mental health was better before quarantine. These results mean that policymakers, educators, and health care providers should urgently develop early intervention programs to improve the wellbeing of children during this unprecedent time. Also, when authorities try to implement restrictions to contain the infection, they should consider the negative effects of these measures on the physical and mental health of children. In future studies, we recommend objective measures of the behavioral changes, so that more accurate estimates of the changes can be obtained. We also recommend a larger representative sample to be taken and to conduct qualitative research such as interviews or focused groups with parents and children. Moreover, variables related to housing such as crowding index, type of housing and monthly income of the family might affect the outcomes measures and should be included in future studies. Also, further studies on the short-term and long-term health consequences of quarantine should be investigated.

## Data Availability Statement

The raw data supporting the conclusions of this article will be made available by the authors, without undue reservation.

## Ethics Statement

The studies involving human participants were reviewed and approved by the Institutional Review Board. They considered this study to be minimal risk to participants and was approved with the IRB # 1632824-2. All participants of this study were provided details about the study objectives, such as participation is voluntary, and no identifiers will be collected. Also, they were informed that submitting the answers to the survey is considered as giving consent to the study. The online consent *via* the online survey was approved by the Ethics Committee. The patients/participants provided their written informed consent to participate in this study.

## Author Contributions

NA-M and ZM conceived, developed the idea, and prepared the questionnaire. NA-M analyzed the data for the project and ZM guided the analysis. NA-M wrote the first draft of the manuscript. ZM was the principal investigator and acted as the mentor for the project. All authors read and approved the final manuscript.

## Funding

The publication of this article was funded by the Medical Education Department at Weill Cornell Medicine-Qatar.

## Conflict of Interest

The authors declare that the research was conducted in the absence of any commercial or financial relationships that could be construed as a potential conflict of interest.

## Publisher's Note

All claims expressed in this article are solely those of the authors and do not necessarily represent those of their affiliated organizations, or those of the publisher, the editors and the reviewers. Any product that may be evaluated in this article, or claim that may be made by its manufacturer, is not guaranteed or endorsed by the publisher.

## Supplementary Material

Supplementary Data Sheet 1 Study instrument.

## Abbreviations

COVID-19: Coronavirus disease 2019
CDC: Centers for Disease Control and Prevention
BMI: Body mass index.
