# Supplementary material for: The Impact of the COVID-19 Lockdown “Home Quarantine” on the Physical Activity and Lifestyle of Children in Qatar
Source: Front Public Health. 2022 May 25;10:877424. doi: 10.3389/fpubh.2022.877424 (PMC9174584; doi:10.3389/fpubh.2022.877424)
Supplement: Supplementary Data Sheet 1 — Study instrument. [file Data_Sheet_1.PDF]

Questionnaire: English Version

1. Age of child (years):
2. Gender of child:
    - Male
    - Female
3. Nationality of child:
4. Current weight of the child (kg):
5. Current height of the child (cm):
6. Relationship of caregiver to child:
    - Mother
    - Father
    - Nanny
    - other (please state the relationship)

7. Rank the child among siblings:
    - Oldest
    - Middle
    - Youngest

8. Age of caregiver:
    - <20
    - 20-29
    - 30-39
    - 40-49
    - >= 50

9. Level of education of caregiver
    - No schooling
    - Primary School
    - Middle School
    - High School
    - Bachelors
    - Masters
    - PhD /Other

10. Select the child's physical activity level before quarantine versus during quarantine (this includes play, games, sports, and planned exercises):

    Before Quarantine:
    - Very High (more than 7 hours per week)
    - High (between 5 and 7 hours per week)

- Moderate (between 2 and 4 hours per week)
- Minimal (less than 2 hours)
- None (0 hours)

During Quarantine:
- Very High (more than 7 hours per week)
- High (between 5 and 7 hours per week)
- Moderate (between 2 and 4 hours per week)
- Minimal (less than 2 hours)
- None (0 hours)

11. On average, how many hours per week did the child do **physical activity** before quarantine versus during quarantine?

Before Quarantine:
- Activity at school during regular hour:
- Activity after school:
- Extra Activity at home:

During Quarantine:
- Online Activity organized by school
- Online Activity after school
- Extra Activity at home

12. Which factors decreased the child's physical activity during quarantine? (Choose all that apply)

- Limited space at home
- Limited equipment
- Child doesn't enjoy it
- Child is alone
- Playgrounds and parks are closed
- Increase in child screen time for school (computers, tablets etc….)
- Increase in child screen time for fun (TV, computers, video games etc….)
- Fear of COVID-19
- Other factors please specify?

13. Which factors increased the child's physical activity during quarantine? (Choose all that apply)

- More time at home
- Encouragement

- Child enjoys activity with family
- Variation between outdoor and indoor activities
- Introduced new activities at home
- I rewarded the child after an activity
- I educated the child (e.g. discussed health benefits)
- Constantly changed exercise plan
- Other factors please specify?

14. On a typical day, how many hours per day does the child spend on screen time for fun activities (e.g. TV, videos, computer, video-games, smartphone, tablet)?

    - **Screen Time Before Quarantine (hours/day):**
        - 0
        - >0 – 1 hour
        - >2 – 3 hours
        - >3 – 4 hours
        - >4 – 5 hours
        - >5 – 6 hours
        - >6 – 7 hours
        - >7 – 8 hours
        - >8 hours

    - **Screen Time During Quarantine (hours/day):**
        - 0
        - >0 – 1 hour
        - >2 – 3 hours
        - >3 – 4 hours
        - >4 – 5 hours
        - >5 – 6 hours
        - >6 – 7 hours
        - >7 – 8 hours
        - >8 hours
15. How many main meals per day did the child consume before quarantine and during quarantine?

    - Before Quarantine (meals/day):
    - During Quarantine (meals/day):

16. Select the amount of unhealthy food (like fast food, soft drinks, sweets etc) the child consumed before quarantine and during quarantine:

    Before Quarantine
    - Never
    - Once per week

- 2 to 4 days per week
- 5 to 6 days per week
- Everyday
- More than once a day

During Quarantine

- Never
- Once per week
- 2 to 4 days per week
- 5 to 6 days per week
- Everyday
- More than once a day

17. Select the amount of healthy food (like vegetables, fruit, whole grains, etc.) the child consumed before quarantine and during quarantine:

Before Quarantine

- Never
- Once per week
- 2 to 4 days per week
- 5 to 6 days per week
- Everyday
- More than once a day

During Quarantine

- Never
- Once per week
- 2 to 4 days per week
- 5 to 6 days per week
- Everyday
- More than once a day

18. Due to the quarantine, the child falls asleep:
    a. Later
    b. Earlier
    c. Same as before the quarantine

19. Due to the quarantine, the child wakes up:
    a. Later
    b. Earlier
    c. Same as before the quarantine

20. How many hours (on average) the child sleeps during the night?

- Before quarantine (hours/night):
- During quarantine (hours/night):

21. Overall, how happy are you with the child's lifestyle during quarantine (this includes physical activity, sleep, food patterns, and screen time)
    - Very happy
    - Happy
    - Neutral
    - Disappointed
    - Very Disappointed

22. How would you rate the mental health wellbeing of your child when comparing it between before and during quarantine?
    - My child mental health was better before the quarantine
    - My child mental health didn't change
    - My child mental health was better after the quarantine.

23. Which of the following best describes your family behavior during the quarantine period?
    - We observed social distancing to a great extent (didn't see relatives or friends)
    - We observed social distancing to some extent (we only saw 1-2 other families)
    - We observed social distancing to lower extent (we saw some relatives and some friends)
    - We didn't observe social distancing at all (we saw everyone we used to see, we didn't change our behavior)

Questionnaire: Arabic Version

1- عمر الطفل (بالسنين):

2- جنس الطفل:

3- جنسية الطفل:
- ذكر
- أنثى

4- وزن الطفل الحالي (كجم):

5- طول الطفل الحالي (سم):

6- العلاقة التي تربطك بالطفل:
  - والدة
  - والد
  - مربّية
  - علاقة أخرى (يرجى تحديد هذه العلاقة)

7- ترتيب الطفل بين أخوته:
  - الكبير
  - الأوسط
  - الصغير

8- عمر الذي يهتم بالطفل
  - أكثر من 20 سنة
  - بين 20 و29 سنة
  - بين 30 و39 سنة
  - بين 40 و49 سنة
  - أكثر من 50 سنة

9- المستوى التعليمي للشخص الذي يهتم بالطفل
  - أميّ
  - تعليم ابتدائي
  - تعليم متوسط
  - تعليم ثانوي
  - شهادة جامعية
  - شهادة دراسات عليا
  - شهادة دكتوراه

10-اختر مستوى النشاط البدني عند الطفل قبل الحجر الصحي وخلاله (بما فيه اللعب، المباريات، التمارين الرياضية وأنشطة بدنية أخرى)

قبل الحجر الصحي:
- عالي جداً (أكثر من 7 ساعات في الأسبوع)

- عالي (بين 5 و7 ساعات في الأسبوع)
- متوسط (بين 2 و4 ساعات في الأسبوع)
- متدني (أقل من ساعتين في الأسبوع)
- لا شيء (0 ساعة)

خلال الحجر الصحي:
- عالي جداً (أكثر من 7 ساعات في الأسبوع)
- عالي (بين 5 و7 ساعات في الأسبوع)
- متوسط (بين 2 و4 ساعات في الأسبوع)
- متدني (أقل من ساعتين في الأسبوع)
- لا شيء (0 ساعة)

11-كمعدّل متوسط، ما هو عدد ساعات النشاط البدني للطفل قبل الحجر الصحي وخلاله؟
قبل الحجر الصحي:
- أنشطة مدرسية خلال ساعات الدوام:
- أنشطة مدرسية بعد الدوام:
- أنشطة إضافية في البيت:

خلال الحجر الصحي:
- أنشطة مدرسية عبر الإنترنت:
- أنشطة ما بعد الدوام عبر الإنترنت:
- أنشطة إضافية في المنزل:

12-ما هي العوامل التي قلّلت من نشاط الطفل البدني خلال الحجر الصحي؟ (اختر كل ما هو ملائم)
- مساحة محدودة في المنزل
- معدات رياضية محدودة
- عدم استمتاع الطفل
- الطفل وحيداً
- إغلاق أماكن اللعب والمتنزهات
- زيادة في عدد ساعات استخدام الطفل للشاشة بهدف الدراسة (كمبيوتر وأجهزة لوحية إلكترونية)
- زيادة في عدد ساعات استخدام الطفل للشاشة بهدف اللعب والمرح (تلفزيون، كمبيوتر، ألعاب فيديو،...)
- الخوف من كوفيد-19
- عوامل أخرى – يرجى ذكرها

13-ما هي العوامل التي زادت من نشاط الطفل البدني خلال الحجر الصحي؟ (اختر كل ما هو ملائم)
- قضاء وقت أطول في المنزل
- التشجيع
- استمتاع الطفل بممارسة الأنشطة مع العائلة
- التنويع بين الأنشطة خارج وداخل المنزل
- ممارسة أنشطة جديدة في المنزل
- مكافأة الطفل بعد ممارسة أي نشاط
- تثقيف الطفل بأهمية ممارسة النشاط البدني (مناقشة الفوائد الصحية)
- تغيير برنامج الأنشطة البدنية باستمرار
- عوامل أخرى – يرجى ذكرها

14-كم ساعة خلال يوم عادي يقضي الطفل أمام الشاشة للّعب والمرح (تلفزيون، فيديو، ألعاب فيديو، هاتف ذكي، جهاز لوحي)؟

- **الوقت أمام الشاشة قبل الحجر الصحي (عدد الساعات في اليوم):**
  - 0 ساعة
  - بين 0 و1 ساعة
  - بين 2 و3 ساعات
  - بين 3 و4 ساعات
  - بين 4 و5 ساعات
  - بين 5 و6 ساعات
  - بين 6 و7 ساعات
  - بين 7 و8 ساعات
  - أكثر من 8 ساعات

- **الوقت أمام الشاشة خلال الحجر الصحي (عدد الساعات في اليوم):**
  - 0 ساعة
  - بين 0 و1 ساعة
  - بين 2 و3 ساعات
  - بين 3 و4 ساعات
  - بين 4 و5 ساعات
  - بين 5 و6 ساعات
  - بين 6 و7 ساعات
  - بين 7 و8 ساعات
  - أكثر من 8 ساعات

15-كم وجبة أساسية كان يتناول الطفل قبل وخلال الحجر الصحي؟
- قبل الحجر الصحي (عدد الوجبات في اليوم):
- خلال الحجر الصحي (عدد الوجبات في اليوم):

16-اختر مقدار الأطعمة غير الصحية التي كان يتناولها الطفل قبل وخلال الحجر الصحي (الوجبات الجاهزة، المشروبات الغازية، الحلويات، ...):

قبل الحجر الصحي:
- ولا مرة
- مرة في الأسبوع
- 2 إلى 4 أيام في السبوع
- 5 إلى 6 أيام في الأسبوع
- يومياً
- أكثر من مرة في اليوم

خلال الحجر الصحي:
- ولا مرة
- مرة في الأسبوع
- 2 إلى 4 أيام في السبوع
- 5 إلى 6 أيام في الأسبوع
- يومياً
- أكثر من مرة في اليوم

17-اختر مقدار الأطعمة الصحية التي كان يتناولها الطفل قبل وخلال الحجر الصحي (الخضروات، الفواكه، الحبوب الكاملة، ...):

قبل الحجر الصحي:

- ولا مرة
- مرة في الأسبوع
- 2 إلى 4 أيام في السبوع
- 5 إلى 6 أيام في الأسبوع
- يومياً
- أكثر من مرة في اليوم

خلال الحجر الصحي:

- ولا مرة
- مرة في الأسبوع
- 2 إلى 4 أيام في السبوع
- 5 إلى 6 أيام في الأسبوع
- يومياً
- أكثر من مرة في اليوم

18-بسبب الحجر الصحي، ينام الطفل:

أ- في وقت متأخر
ب- باكراً
ت- في نفس التوقيت قبل الحجر الصحي

19-بسبب الحجر الصحي، يستيقظ الطفل:

أ- في وقت متأخر
ب- باكراً
ت- في نفس التوقيت قبل الحجر الصحي

20-كم ساعة (كمعدل متوسط) ينام الطفل في الليل؟

- قبل الحجر الصحي (عدد الساعات في الليل):
- خلال الحجر الصحي (عدد الساعات في الليل):

21-بشكل عام، ما مدى رضاك عن نمط الحياة الذي يتبعه طفلك خلال الحجر الصحي (بما فيه الأنشطة البدنية، ساعات النوم، الأكل، استخدام الشاشة)

- سعيد جداً
- سعيد
- محايد
- خائب الأمل
- خائب الأمل جداً

22-كيف تقيّم صحة طفلك النفسية ورفاهيته في حال قارنتها بفترة ما قبل الحجر وخلاله؟

- صحة طفلي النفسية كانت أفضل قبل الحجر الصحي
- صحة طفلي النفسية لم تتغيّر

- صحة طفلي النفسية أفضل بعد الحجر المنزلي

23-أي من الأمور التالية تصف تصرفات عائلتك خلال فترة الحجر الصحي؟

- طبّقنا التباعد الاجتماعي إلى أقصى حدود (لم نجتمع بالأقارب والأصحاب)
- طبّقنا التباعد الاجتماعي إلى حدّ ما (اجتمعنا بعائلة أو عائلتين)
- طبّقنا التباعد الاجتماعي بدرجة قليلة (اجتمعنا ببعض الأقارب والأصدقاء)
- لم نطبّق التباعد الاجتماعي على الإطلاق (اجتمعنا بطل الأشخاص الذين تعوّدنا رؤيتهم، لم نغيّر من تصرفاتنا)
